# An investigation of objective and subjective types of binge eating episodes in a clinical sample of people with co-morbid obesity

**DOI:** 10.1186/2050-2974-1-26

**Published:** 2013-08-08

**Authors:** Marly Amorim Palavras, Christina Marcondes Morgan, Ferrari Maria Beatriz Borges, Angélica Medeiros Claudino, Phillipa J Hay

**Affiliations:** 1 Programa de Atenção aos Transtornos Alimentares (PROATA), Department of Psychiatry, Universidade Federal de São Paulo (UNIFESP), São Paulo, SP, Brazil; 2 Sociedade Brasileira de Psicologia Analítica, São Paulo, SP, Brazil; 3 Centre for Health Research School of Medicine University of Western Sydney, Psychiatry James Cook University, Sydney, Australia; 4 University of Western Sydney, Locked Bag 1797, Penrith NSW 2751, Australia

**Keywords:** Binge eating, Diagnostic criteria, Objective binge eating episodes, Subjective binge eating episodes

## Abstract

**Background:**

Objective binge eating episodes (OBEs) refer to binge eating on an unusually large amount of food and are the core symptom in current definitions of bulimia nervosa (BN) and binge eating disorder (BED). Subjective binge eating episodes (SBEs) refer to eating on a small or moderate amount of food (that is perceived as large) and like OBEs are associated with loss of control (LOC). Reaching consensus on what is considered a large amount of food can however be problematic and it remains unclear if the size of a binge is an essential component for defining a binge eating episode. The aim of this study was to compare the eating disorder features and general psychopathology of subjects reporting OBEs with those reporting only SBEs.

**Methods:**

This is a retrospective secondary analysis of data from 70 obese participants at the recruitment phase of a multicentre trial for BED. Individuals who answered positively to the presence of binge eating and LOC over eating had their binge eating episodes further explored by interview and self-report. Two groups, those who reported current OBEs (with or without SBEs) and those who reported current SBEs only were compared for age, gender, marital status, body mass index (BMI), indicators of LOC over eating, severity of binge-eating and associated psychopathology.

**Results:**

The majority of participants in both the OBE and SBE groups endorsed the experience of at least four indicators of LOC. There were no significant differences between the groups. Both groups had high levels of binge-eating severity, moderate severity of associated depressive symptoms and frequent psychiatric co-morbidity.

**Conclusion:**

Treatment seeking participants with obesity who reported SBEs alone were similar to those who reported OBEs in terms of eating disorder features and general psychopathology. These findings suggest that classificatory systems of mental illnesses should consider introducing SBEs as a feature of the diagnostic criteria for binge eating and, thus, facilitate the inclusion of participants with SBEs in treatment trials.

## Background

Binge eating is currently defined as a discrete episode of overeating of an objectively large amount of food associated with a feeling of loss of control (LOC). It is the core symptom of bulimia nervosa (BN) and binge eating disorder (BED) and it can be present in purging-type anorexia nervosa (AN) [1]. In addition, three of a set of five criteria that are markers of LOC are required for the definition of an objective binge eating episode (OBE) in the diagnosis of BED in the Diagnostic and Statistical Manual of Mental Disorders, 4^th^ edition, text revised (DSM-IV-TR) [1]. The subjective binge eating episode (SBE) is defined where LOC is also experienced but the amount of food consumed is small or moderate, although perceived as large [2].

To date, the presence of SBEs has not been specifically included in the diagnostic criteria for binge eating related disorders [1] and people with SBEs alone are thereby not included in treatment trials. However, there is ample evidence of the frequent occurrence of SBEs with or without concurrent OBEs in community samples [3-5] and in clinical samples [6-10]. For example, in a recent interview-based study with female college students, 11.8% of the participants reported the presence of SBEs, 15.3% had OBEs and 13.6% described both, OBEs and SBEs [4]. Another investigation in 339 adult women with eating disorders (ED) features recruited from the general community reported current prevalence of 58.4% for weekly SBEs and of 41.5% for weekly OBEs [5]. One study in a clinical sample reported prevalence of 11.8% for presence of SBEs alone, 30.5% for presence of OBEs alone and 44% for both [6]. In addition, research in children and adolescents has replicated the results obtained in adult samples [11,12]: in a non-clinical sample, nineteen children (13.3%) reported episodes of LOC over eating (9 with SBE and 10 with OBE) in the previous 28 days [13]. Finally, data from an adolescent community sample showed that 9.3% of the subjects reported SBEs, 4.8% reported OBEs and 2.6% reported both SBEs and OBEs in the same time frame [14].

The definition of what is considered a large amount of food can also be problematic. A recent systematic review synthesizes the existence of a broad variation of caloric intake during a binge episode [15]. The lay use of the term “binge eating” does not always correspond to the clinical one and may include the ingestion of forbidden foods with excessive calories [16], or intake of small amounts of food (for example, fewer than 500 kcal) which are perceived as large [17]. Seminal research found that when not prompted, the participants defined a binge only on the basis of the consumption of a large amount of food. However when asked to choose between eleven features, the preferred symptoms were extreme fullness, LOC and dysphoric mood as well as the large amount of food eaten [3]. Some studies have also reported that individuals with BED and BN identify binge episodes more by feelings of LOC than by the amount of food eaten, although those with BN tend to report larger binge episodes [15,18-20]. Because of the difficulty in operationalising the concept of a “large amount”, it has been proposed that LOC should be the defining feature of a binge eating episode [4,14,15,21,22].

A further issue is that many studies do not support differences between people with SBEs and people with OBEs in regards to ED psychopathology such as antecedents of binge eating (e.g. boredom, cravings), compensatory behaviors (e.g. self-induced vomiting), eating patterns (e.g. food avoidance, restraint) and weight and shape concerns [10,23,24]. In addition, people with either OBEs or SBEs have been found to have similar levels of general psychopathology (depression, anxiety and stress), as well as functional impairment [4,10,22,24-26]. Peterson et al. [6] have also reported high levels of instability in SBEs and OBEs over time.

It remains unclear if the size of a binge, or other markers of LOC, are essential components for defining a binge eating episode, with meaningful validity and clinical utility. Colles et al. (2008) investigated the LOC indicators in two groups of bariatric surgery candidates - those with full BED and those with “Subjective LOC”. They found fewer participants in the second group endorsed the item “eating alone”, and this item was also the least frequently endorsed criteria in the subjective LOC group [25]. Very recent research examined the predictive power of these indicators for the diagnosis of BED. The best positive predictive power of the five items was found for “eating alone” (PPP = 0.80) and “feeling disgusted, guilty or depressed after binge eating” had the best negative predictive power (NPP = 0.93). These two items had the best sensitivity and specificity respectively [27].

The aim of this study was thus to examine whether participants with overeating and co-morbid obesity reporting SBEs alone or OBEs (with or without SBEs) differ in terms of sociodemographics and clinical characteristics including BMI, severity of binge eating, presence of markers of LOC and associated general psychopathology. Our specific hypothesis was that there would be no difference in the endorsement of diagnostic criteria for markers of LOC.

## Methods

### Design and participants

This is a retrospective secondary analysis of data collected at the screening phase (first visit to the site) of a clinical trial testing a pharmacological treatment for BED at the Eating Disorders Program (PROATA) of the Federal University of São Paulo, Brazil. This trial was approved by the human ethics committee of the university.

The sample selected for the present study was not determined by whether the participants were selected for the pharmacological trial (original study) or not. For this study, the inclusion criteria were: adults (between 18 and 60 years) of both genders, obese (body mass index [BMI]: weight [kg]/height [m^2^] ≥ 30 kg/m^2^) and reporting recurrent episodes of binge eating. For the interest of this study, we selected participants that answered positively to an initial screening containing three questions about: a) eating a large amount of food, b) eating an amount larger than most people would eat considering the circumstances and c) describing a sense of LOC during the episode. As the Structured Clinical Interview for DSM-IV Axis I Disorders-Patient Edition (SCID-I/P) was used as a study measure in the original trial, two items were also considered for the inclusion of participants in this study: the first question of the BN module that investigates the presence of binge eating, where only item A2 – LOC – was needed to score positively), and item D of the BED module (also scoring positively either with a threshold or subthreshold score). Thus, the sample considered for this study could include volunteers who did not meet full inclusion criteria for the original trial (see details in Claudino et al.) [28]. For instance, participants could be included if they reported recurrent LOC over eating associated with eating large amounts of food (OBE) or not (SBE) and with frequency/duration meeting DSM-IV criteria (twice a week for six months – criteria for BED) or lower (at least once a week for six months). For this study the exclusionary criteria were being illiterate, not being able to give accurate responses to questionnaires, a concurrent severe psychiatric illness (e.g. psychosis) or meeting DSM-IV-TR criteria for AN or BN.

### Procedures

During the screening phase of the trial, participants underwent measurement of height and weight as well as interviews and questionnaires that evaluated eating behaviors, symptoms of depression and psychiatric morbidity, after signing written informed consent.

Participants who reported recurrent binge eating episodes at the initial screening and at the SCID-I/P were invited to participate in a further investigation (optional) in which a structured interview developed by the authors (CMM & MBFB) was used. This was composed of questions designed to determine the size of their typical binge eating episode (Appendix). A detailed description of a recent typical episode of binge eating and information about context, size and the duration of the episode was required. If the episode did not involve an objective large amount of food, participants were asked about a second episode of a larger amount of food. All the participants’ binge eating reports were evaluated separately by two researchers (MAP and CMM) with experience in ED diagnosis and treatment. These assessors were instructed to assess the binge episodes according to the DSM-IV-TR [1] criteria for OBE and the EDE [2] definitions of OBE and SBE. This included the discrimination between SBEs and OBEs based on the amount eaten (large for OBE and small or moderate for SBE) plus the sense of LOC. If the two researchers disagreed in the evaluation of a report, a discussion was proposed for a consensus, and if the disagreement remained, a third specialist (AMC) was consulted.

Eighty seven people who reported recurrent binge eating in the screening phase in our site completed this additional questionnaire where a more detailed description of the binges was asked. From these participants, 17 were excluded from this sample for the following reasons: 12 (13.8%) could not give accurate and reliable descriptions of binges, 1 (1.1%) had incomplete data at SCID-I/P and 4 (4.6%) did not meet criteria for sense of LOC over eating (negative answer to item A2 of BN module) and/or periodicity (negative answer to item D of BED module) in the SCID-I/P. The seventy remaining (80.4%) were included in this study.

### Assessment instruments

#### Weight status

Weight and height were assessed by a calibrated balance beam scales and stadiometers.

#### Descriptive questionnaire for Binge Eating Episodes

This interview aimed at collecting a more detailed description of typical episodes of binge eating for the individual. It consists of a first question investigating the date, the occasion (routine or special situation), the hour, the place, with whom and a detailed description of a typical binge eating episode. If this first description did not include an OBE, a second question asked about any other binge eating episode larger than the first one with the same topics mentioned above.

#### Structured Clinical Interview for DSM-IV Axis I Disorders-Patient Edition (SCID-I/P)

This reliable and valid structured interview for psychiatric diagnoses according to the DSM-IV was employed for the BED diagnoses and evaluation of the presence of recurrent episodes of eating a large amount of food when feeling out of control over eating [29].

#### Mini-International Neuropsychiatric Interview (MINI Plus)

This short, well-validated structured diagnostic interview was used for evaluation of associated psychiatric morbidities [30]. The MINI Plus does not evaluate BED (only AN and BN).

#### Binge Eating Scale

The Binge Eating Scale (BES) is a widely used 16-item self-report measure which evaluates the binge eating episodes severity. A score of 0–17 was considered “absent”; 18–26 “moderate”; and ≥ 27 “severe” [31,32].

#### Beck Depression Inventory

The Beck Depression Inventory (BDI) is a well-validated 21-item self report inventory used for measuring the severity of depressive symptoms. A score of 0–9 was considered “normal”; 10–18 “mild to moderate”; 19–29 “moderate to severe”; and 30–63 “severe” [33].

### Statistical analysis

Descriptive data are reported as mean (SD) for continuous data and n (%) for categorical data. T-tests were performed for continuous data and Chi-Square tests and Fisher Exact test respectively for categorical data. Differences between groups were considered significant when p values were < .05.

## Results

Participants were categorized in two groups: those who described a typical binge eating episode that involved an actual large amount of food (OBE group, n = 56), and those who described the typical binge eating episode as a binge that involved an amount of food perceived as large by the subject - but not considered large by the examiners - and who denied ever having experienced larger episodes (SBE group, n = 14).

The final sample was composed of 70 adults with mean age (SD) of 36.9 years (11.05) and mean (SD) BMI of 37.20 (3.86) kg/m^2^. There were no significant differences between groups for age, sex, race and marital status (Table 1).

**Table 1:** **Sociodemographic characteristics of the Objective Binge Eating Episode** (**OBE**) **and Subjective Binge Eating Episode** (**SBE**) **groups**

	**Total n =70**	**OBE n= 56 (80%)**	**SBE n = 14 (20%)**	**p (OBE *vs *** **SBE)**
Age/years				
Mean (SD)	36.9 (11.05)	36.3 (10.90)	39.5 (11.67)	0.326^c^
Sex				
n (% female)	66 (94.3)	53 (95)	13 (93)	1.000^d^
Race				
n (% white)^a^	44 (66.7)	38 (72)	06 (46)	0.105^d^
Marital Status				
n (% married)^b^	34 (49.3)	29 (55)	05 (42)	0.414^e^

^a^For race: OBE (n = 53); SBE(n = 13); ^b^For marital status: OBE(n = 53); SBE(n = 12); ^c^t-Test; ^d^Fisher Exact Test; ^e^Chi-square Test.

As shown in Table 2, SBE and OBE groups did not differ in terms of severity of binge eating behavior (BES scores), severity of depressive symptomatology (BDI scores), presence of associated psychiatric morbidities and degree of obesity. The two groups presented similar results in the BES, with a mean score higher than 27 points, which indicates a high severity of binge eating behavior and related feelings and attitudes. Similarly, scores on the BDI for both groups indicated moderate severity of associated depressive symptomatology. The results obtained with the comparison using the interview MINI-Plus showed a higher percentage of the OBEs participants reporting at least one psychiatric comorbidity compared to SBEs participants, but the difference was not significant. The majority of the participants in both groups endorsed the experience of at least four indicators of LOC over eating (DSM-IV-TR criterion B for BED) evaluated with the SCID-I/P. On the “Eating Alone” item, a smaller percentage of SBEs individuals confirmed eating alone, although this difference did not reach statistical significance.

**Table 2:** **Comparative clinical features of the Objective Binge Eating Episode** (**OBE**) **and Subjective Binge Eating Episode** (**SBE**) **groups**

	**SBE (n = 14)**	**OBE (n = 56)**	**p**
	**Mean (SD)**	**Mean (SD)**	
Binge Eating Scale	30.21 (6.72)	31.32 (7.47)	0.615^h^
Beck Depression Inventory^a^	20.00 (7.98)	21.57 (10.30)	0.621^h^
Body Mass Index (kg/m^2^)	37.81 (4.48)	37.05 (3.72)	0.512^h^
	n (%)	n (%)	
Mini International Neuropsychiatric Interview-Plus^b^ (with at least one morbidity)	6 (50)	35 (71)	0.183^i^
Presence of markers of loss of control			
according to Structured Clinical Interview for DSM-IV, Axis I, Patient edition			
Rapid eating^c^	11 (84.6)	48 (88.9)	0.647^i^
Eating until uncomfortably full^d^	14 (100)	49 (90.7)	0.575^i^
Eating when not hungry^e^	14 (100)	51 (98.1)	1.000^i^
Eating alone^f^	6 (42.9)	37 (68.5)	0.076^j^
Feeling guilty^g^	14 (100)	48 (90.6)	0.576^i^

^a^BDI: SBE (n = 12); ^b^MINI-Plus: OBE(n = 49); SBE(n = 12); ^c^Rapid eating: OBE(n = 54); SBE(n = 13); ^d^Eating until uncomfortably full: OBE(n = 54); ^e^Eating when not hungry: OBE(n = 52); ^f^Eating alone: OBE(n = 54); ^g^Feeling guilty: OBE(n = 53); ^h^t-Test; ^i^Fisher Exact Test; ^j^Chi-square Test.

## Discussion

The current study investigated, in a clinical sample, the potential differences between people with obesity and SBEs (only) or OBEs (with or without SBEs) in terms of sociodemographics and clinical profile, including eating disorder and general psychopathology. The results indicated a strong similarity between both groups in all variables. Both groups showed a high severity of binge eating, similar endorsement of the DSM-IV-TR indicators of LOC over eating, one of the diagnostic features of BED (criterion B), moderate levels of depressive symptomatology and high levels of co-morbidity. Our findings support results from previous studies which suggest that individuals that engage only in SBEs do show an eating disturbance that is associated with eating-related and general psychopathology [4,14,24-26,34].

This study supports research reports of participants with bulimic-type ED [5] and of bariatric surgery patients with and without BED [25] that have also found similar sociodemographic profiles for people with OBEs people with SBEs. In addition, Keel et al. (2001) found few differences between an OBE group and a SBE group, both with BN; however the OBE group was older [34].

Interestingly, our BMI findings support a similar impact of both OBEs and SBEs on body weight, as both groups showed mean weights in the moderate severity level of obesity. In a BN study, there were no differences between the type of binge and the BMI measures [10]. Different findings were found in another study with participants with bulimic-type ED, in which the OBE group had a higher weight than SBE group [5]. A study with bariatric surgery candidates showed a higher mean BMI in the OBE group compared to the SBE group, which was also higher than the non binge-eating group; however the authors found that despite the absence of large amounts of food eaten, individuals with SBEs were at risk of obesity and weight gain, although the mechanism was not clear [25].

In relation to the psychopathological profile of our sample, both groups showed clinically significant severity of binge eating and depressive symptoms, as indicated by BES and BDI scores in the high and moderate ranges, respectively. In addition, our results showed no statistical difference in percentage of at least one co-morbid DSM-IV-TR psychiatric diagnosis (based on MINI-Plus interview). However, over two thirds (71%) of participants in the OBE group had at least one associated psychiatric diagnosis, compared to half of the SBE group. Latner et al. (2007), in a study with participants with bulimic type ED, found similar results for both, the SBE and the OBE groups in terms of general psychopathology including depression, stress and anxiety measured by DASS (Depression Anxiety Stress Scale) and reported that “… both episode types significantly and independently predicted global eating disorder psychopathology” (page 2208). In addition, these authors also commented that SBEs may also be considered a marker of psychopathology [24]. Similar results have been found in several other studies [4,9,10,25,34]. In contrast, OBEs, but not SBEs, have been associated with impulsiveness in women with bulimic-type ED [34].

Our findings do not support differences between the groups in endorsement of the five diagnostic indicators of LOC over eating (criterion B for BED) of DSM IV-TR and are consistent with those of another study [25]. The majority of participants in both groups answered positively to the features that indicate LOC over eating during a binge. Thus these indicators appear to be both common and independent of size of eating episode. There was however a statistical trend for the OBE group to be more likely to endorse ‘eating alone’ than those in the SBE. This finding needs further investigation in a larger sample.

At this stage, little is known with regard to how much the treatments applied to those suffering of OBEs (BN and BED) can be recommended and efficacious for those with only SBEs. One study of CBT resulted in remission of OBEs but only reduced SBEs [35]. SBEs may not respond to early treatment, possibly due to the increased presence of cognitive distortion [9,36]. Two new trials reinforced the importance of the treatment for SBE group with a focus more on cognitive distortions and negative affect than on the over-eating issues [37,38]. The specific management of SBEs as compared to OBEs requires more research.

The main limitations of the current study are its retrospective cross-sectional design and the low numbers of the SBE group (and thus unbalanced group sizes), which may have restricted the power to detect differences between groups. However, notwithstanding the lack of significance, no clinically relevant differences between groups were identified. Another limitation of the study is that the report of binge eating size was based on interview questions designed to ensure OBEs were present as eligibility criteria for a randomized controlled trial. Also, the OBE group could include participants with SBEs, thus comparisons in this study are not based on a sample of people with “pure” OBEs versus a group with “pure” SBEs. This may have overestimated the pathology of the SBE group and underestimated the pathology of the OBE. Further limitations are the under-representation of men, the use of self-report instruments to evaluate the severity of the compulsive eating and depressive symptomatology, and that missing data may lead to heterogeneous comparisons. The use of instruments which allow a better exploration of the eating patterns, as the Eating Disorder Examination [2] would have been preferable. It is important to acknowledge the preliminary nature of this study and its limitations, especially the small sample size. Thus conclusions should be regarded as tentative. Finally, although written prior to the revision of the DSM-5 [39] the findings remain relevant as the DSM-5 criteria have not changed in regards to the non-inclusion of SBEs and the five diagnostic indicators of LOC in BED are retained.

## Conclusion

The current diagnostic systems for classification of ED should consider including SBEs for diagnosing individuals with BED. This inclusion might possibly reduce the number of individuals diagnosed with an Eating Disorders Not Otherwise Specified (EDNOS) or not receiving an ED diagnosis and allow for an increased number of such individuals being included in treatment trials. The definition of the binge episode could be further improved by strengthening the importance of the sense of LOC and mentioning the “perception” of eating a large amount of food, irrespective of whether the amount of food eaten is really large. Finally, our findings highlight the need for clinicians to assess the presence of SBE and its clinical impact.

## Appendix

### Descriptive Questionnaire for Binge Eating Episodes

Christina Marcondes Morgan and Maria Beatriz Ferrari Borges

**Disclaimer**: This has been translated from Portuguese and is not intended for use in this form but intended for the information of readers.

Now I would like to ask you some questions about these episodes in which you feel you lose control and eat large amounts of food, these are called binge eating episodes.

1) Please, try to remember the latest typical episode when you felt a sense of loss of control and ate an amount of food that most people would consider excessive.

a) When did it happen (date)? …………………………………………………………………..

b) Was it a special occasion or a routine day?

c) If a routine day: Any important event?………………………………………..

d) If a special occasion: Which one?

e) What time was it? ……………………… Where were you? …………………………

f) Who were you with?

e) Now, I would like you describe to me all you ate during this episode, including drinks and desserts. (*Record everything that the participant says*, *without interrupting*. *Then*, *read aloud the food list and ask if any item is missing*. *Verify if the participant included drinks and desserts*. *Finally*, *go back to each item and ask about its preparation and the amount eaten*. *Ask the participant to use the food models offered by the interviewer* (*book*) *to define the amount*. *If the participant reports he*/*she spent the* “*whole day eating*, *ask him*/*her to define the period when****the loss of control was more relevant***.

2) (If *the description of the episode provided does not include an objectively large amount of food*). Are there other episodes when you eat an amount of food that is larger than then the previously described? ( ) Yes ( ) No Please, describe one of these episodes.

a) When did it happen (date)? ……………………………………………………………

b) Was it a special occasion or a routine day?

c) If a routine day: Any important event on that day?………………………………………..

d) If a special occasion: Which one?………………………………………..

e) What time was it? ……………………… Where were you? ……………………………….

f) Who were you with?

e) Now, I would like you to describe to me everything that you ate during this episode, including drinks and desserts. (*Follow the guidelines of the previous description*).

## Abbreviations

AN: Anorexia Nervosa; BDI: Beck Depression Inventory; BED: Binge Eating Disorder; BES: Binge Eating Scale; BMI: Body Mass Index; BN: Bulimia Nervosa; CBT: Cognitive Behavioral Therapy; DASS: Depression Anxiety Stress Scale; DSM-IV-TR: Diagnostic and Statistical Manual of Mental Disorders Fourth Edition-Text Revised; ED: Eating Disorder(s); EDNOS: Eating Disorder Not Otherwise Specified; ICD: International Classification of Diseases; LOC: Loss of Control; MINI Plus: Mini-International Neuropsychiatric Interview; OBE: Objective Binge Eating; SBE: Subjective Binge Eating; SCID I/P: Structured Clinical Interview for DSM-IV Axis I Disorders-Patient Edition.

## Competing interests

AMC and PH are members of the World Health Organization Working Group on Feeding and Eating Disorders for the Revision of ICD-10 Mental and Behavioral Disorders and this paper represents personal views of the authors.

## Authors’ contributions

MP, CM, MB, AC were responsible for participant recruitment and assessment. MP carried out the statistical analyses. MP, AC and PH conceived of the study, and participated in its design and coordination and helped to draft the manuscript. All authors read and approved the final manuscript.
